# Xome-Blender: A novel cancer genome simulator

**DOI:** 10.1371/journal.pone.0194472

**Published:** 2018-04-05

**Authors:** Roberto Semeraro, Valerio Orlandini, Alberto Magi

**Affiliations:** 1 Department of Experimental and Clinical Medicine, University of Florence, Florence, Italy; 2 Medical Genetics Unit, Meyer Children’s University Hospital, Florence, Italy; Pennsylvania State University, UNITED STATES

## Abstract

The adoption of next generation sequencing based methods in cancer research allowed for the investigation of the complex genetic structure of tumor samples. In the last few years, considerable importance was given to the research of somatic variants and several computational approaches were developed for this purpose. Despite continuous improvements to these programs, the validation of their results it’s a hard challenge due to multiple sources of error. To overcome this drawback different simulation approaches are used to generate synthetic samples but they are often based on the addition of artificial mutations that mimic the complexity of genomic variations. For these reasons, we developed a novel software, Xome-Blender, that generates synthetic cancer genomes with user defined features such as the number of subclones, the number of somatic variants and the presence of copy number alterations (CNAs), without the addition of any synthetic element. The singularity of our method is the “morphological approach” used to generate mutation events. To demonstrate the power of our tool we used it to address the hard challenge of evaluating the performance of nine state-of-the-art somatic variant calling methods for small and large variants (VarScan2, MuTect, Shimmer, BCFtools, Strelka, EXCAVATOR2, Control-FREEC and CopywriteR). Through these analyses we observed that by using Xome-Blender data it is possible to appraise small differences between their performance and we have designated VarScan2 and EXCAVATOR2 as best tool for this kind of applications. Xome-Blender is unix-based, licensed under the GPLv3 and freely available at https://github.com/rsemeraro/XomeBlender.

## Introduction

The advent of NGS technologies and the parallel development of powerful computational tools, have drastically modified the biological and biomedical research over the past several years.

Particular advantages from these changes have been gathered by cancer research, where the complex landscapes of somatic variants have been investigated in a wide variety of tumor types [1]. In the last few years, cancer genome projects have been properly devised to catalogue the diversity of DNA mutations present in different cancers via high throughput DNA sequencing of matched tumor-normal samples.

The principal aims of these projects are: (i) finding correlations between mutation profiles and clinical outcomes and (ii) identify mutations driving cancer progression and targets for novel therapeutic developments [2]. The discovery of these mutations, also called somatic variants, consists in finding variant alleles that are present in the tumor but not in the germline cells [3]. By using this procedure, it is possible to identify single nucleotide variants (SNVs) [4, 5], small insertions and deletions (InDels) [6] and structural variants [7, 8]. However, proper identification of DNA mutations relies on the sequencing technology, data quality, and statistical methods used to analyze sequencing data [9]. In fact, despite the development of many bioinformatics pipelines and much effort to give standard rules for the assessment of good quality NGS experiments [10], no clear guidelines exist on how to practically analyze genome-sequencing data, as the optimization of somatic variant identification constitutes an important challenge in computational biology [11].

In recent years, several methods have been developed to discover somatic mutation [12–16], however, the validation of the results produced by these methods on real data is an hard challenge because no tumor genome has been completely characterized (i.e., with all real somatic mutations known). As a consequence, defining a gold standard for somatic mutation detection is fraught with challenges due to various sources of error such as artifacts occurring during PCR amplification or sequencing errors, incorrect local alignments of reads, tumor heterogeneity and sample contamination.

To overcome the lack of fully characterized tumor genomes several works proposed the use of simulation approaches to create synthetically mutated genomes by using reads simulation or downsampling of real sequencing data [12, 17]. In the first approach, reads are simulated on the basis of a probabilistic model and then enriched with “spiked-in” mutations [18]. Even if some of these methods are very sophisticated, they are not capable to model the full diversity of nonrandom sequencing errors. In downsampling, reads are randomly drawn from real sequencing data and used to create a new subsample by adding mutations at a desired allelic fraction (AF) [17]. Although these approaches are capable to simulate multiple subclones and sample contamination preserving the error profile of sequencing technologies, synthetic mutations can not completely mimic the complexity of genomic variations due to unpredictability of error events. For these reasons, we developed a novel software, Xome-Blender, that generates synthetic cancer genomes with user defined features such as the number of subclones, the number of somatic variants and the presence of CNAs, without the addition of any synthetic element.

The key idea at the base of our tool consists in using real sequencing data of normal individuals and subtracting reads that contain alternative alleles in heterozygous variants loci. By using this recipe, at each step of variants removal, we move backwards in tumor evolution process generating a subclone that is the progenitor of the starting sample. All the subclones are then subsampled at the desired fraction and merged to obtain a synthetic heterogeneous tumor sample matching the user requirements. In this way it is possible to simulate somatic SNVs and InDels with the desired AF. Moreover, exploiting a strategy based on adding or removal of reads, Xome-Blender is also able to generate somatic CNAs (sCNAs) of predefined length without altering the AF of single nucleotide and InDel variants.

In this work we demonstrated the power of our tool in generating tumor samples with different subclonal architectures and we used it to address the hard challenge of evaluating the performance of nine state-of-the-art somatic variant calling methods for small and large variants.

## Results

### Overview of current approaches

Computer simulation of genomic data has become increasingly popular for assessing and validating biological models or for gaining an understanding of specific data sets [19]. Improved computational methods and more efficient bioinformatic tools are constantly developed to provide faster processing and more accurate inferences. However, it is essential that these methods be benchmarked against existing tools with similar functionality, to show their superiority in at least some aspect.

In general, computational methods can be benchmarked using experimental and/or simulated genomic data, and although validation with experimental data is essential as it represents real scenarios, owing to the lack of complete knowledge on real experiments, it is very challenging to use them for the assessment of algorithm performance.

Alternatively, in silico simulations allow us to generate as much data as desired, under controlled scenarios and with predefined parameters for which the true values are known, thus nicely complementing validation with real data [20]. In the last few years, simulation approaches have gained ground in cancer genomics due to the lack of guidelines for the assessment of good quality NGS experiments. In fact, despite the development of many bioinformatics pipelines and much effort to give standard rules to practically analyze tumor genome sequencing data, the error proneness of the sequencing platforms leads to the generation of biased reads that could invalidate the products of the analysis, making very hard applications such as the somatic variant calling. In fact, calling somatic variants is a harder problem than calling germline variants [21] because of variability in the number of somatic mutations, extent of tumor subclonality and effects of copy number alterations.

At present, computational methods for simulating cancer sequencing data can be classified in two main approaches: those based on synthetic reads generation and those on downsampling of real sequencing data. NGS reads simulators are based on probabilistic models that allow to generate sequences with “spiked-in” mutations and that mimic the attributes of different sequencing technologies, such as read length and error rate. These tools differ in several aspects, such as sequencing technology they simulate, input requirements or output format, but have several aspects in common. With few exceptions, all programs need a reference sequence (that can be a particular genomic region, multiple genomic regions that are concatenated, a chromosome or a complete genome.) and multiple parameter values that indicate the technological (i.e., insert sizes, read lengths, error rates and quality scores) and/or the biological (i.e., GC content, InDel rates and substitution rates) features of the sequencing experiment to be simulated. The reads produced by these tools can show user defined features such as type, length and number according to the sequencing technology assumed and the desired coverage. The read type can be specified directly or indirectly by defining particular insert sizes. By default, most simulators assume single-end reads [19]. Apart from sequencing errors, many tools can also introduce different types of genomic variants in the simulated reads [22], such as SNVs, InDels, inversions, translocations, copy number variants (CNVs) and short tandem repeats (STRs).

The general strategy is to create a mutated sequence by introducing genomic variants in the reference sequence before the generation of reads. In most cases, these variants are simulated using a given mutation rate, so the mutated sequence differs by a given percentage from the reference sequence; however, programs like DWGSIM [4] and EAGLE [23] require a file with known mutations (in plain text, variant call format (VCF) or browser extensible data (BED)-like format).

Some programs are capable of generating population level diversity by creating several mutated sequences from a single reference sequence. Programs like GemSim [24] and Mason [25] can generate sets of related haplotypes differing by at least one SNV from the reference sequence. In GemSim, users may also create their own tab-delimited haplotype file providing the specific position and mutation introduced. Although these methods are based on very sophisticated probabilistic models and allow to generate very complex genomic alterations, they are not capable to reproduce the nonrandom component of error profiles of real sequencing data.

In order to overcome the limits of synthetic sequences simulators, Cibulskis et al. [12] were the first to use the downsampling approach to create simulated dataset by randomly drawing reads from real sequencing data. The idea at the base of its approach is to exploit real cancer sequencing data with validated somatic mutations and randomly excluding reads from the original data until a desired depth of coverage is reached. Although the error profile of sequencing technologies are preserved, the repertoire and AF of mutations is limited to examples previously detected, and this approach can not reproduce intra-tumor heterogeneity and sample contamination.

More recently, Ewing et al. [17] developed a novel tool (BAMSurgeon) that takes the sequencing data of a normal sample as input and it randomly adds mutations at a desired AF. Although it allows the simulation of multiple subclones and sample contamination, the adding of synthetic mutations can not completely mimic the complexity of genomic variations due to unpredictability of error events.

At present, few publicly available tools can be exploited for the simulation of cancer genomes (see Table 1) and these include methods based on both downsampling or synthetic reads approaches. Although these softwares are capable to simulate several tumor genomic features, none of them allow to produce complex cancer samples with subclonal architectures and avoided by synthetic elements. tHAPMix, IntSIM and BAMSurgeon have automatic pipelines for the simulation of tumor subclonal architectures, while the other tools can perform this task by creating and merging different simulated samples. The great majority of these methods allow to only simulate small somatic variants (SNVs and InDels) with the exception of SCNVSim, EAGLE, IntSIM and BAMSurgeon that are capable to generate structural variants or CNAs only. Moreover, in all these methods, simulated variants are based on the addition of synthetic elements that do not mimic the sequencing technologies biases. Finally, only tHAPMix and BAMSurgeon are able to produce results in.bam format, while the other tools output reads in fasta/fastQ formats that need to be aligned against a reference genome.

**Table 1:** Cancer simulation tools.

	Variants			Output	
	SNVs	InDels	CNAs	Het	FO
BAMSurgeon	Yes	Yes	No	Yes	BAM
DWGSIM	Yes	Yes	No	No	FQ
EAGLE	Yes	Yes	Yes	No	FQ
GemSim	Yes	Yes	No	No	FQ
IntSIM	Yes	Yes	Yes	Yes	FQ
Mason	Yes	Yes	No	No	FQ,FA
SCNVSim	No	No	Yes	Yes	FA
tHapMix	Yes	Yes	Yes	Yes	BAM

FA, FASTA; FO, format; FQ, FASTQ; Het, Heterogeneity.

### The tool

**Fig 1:**
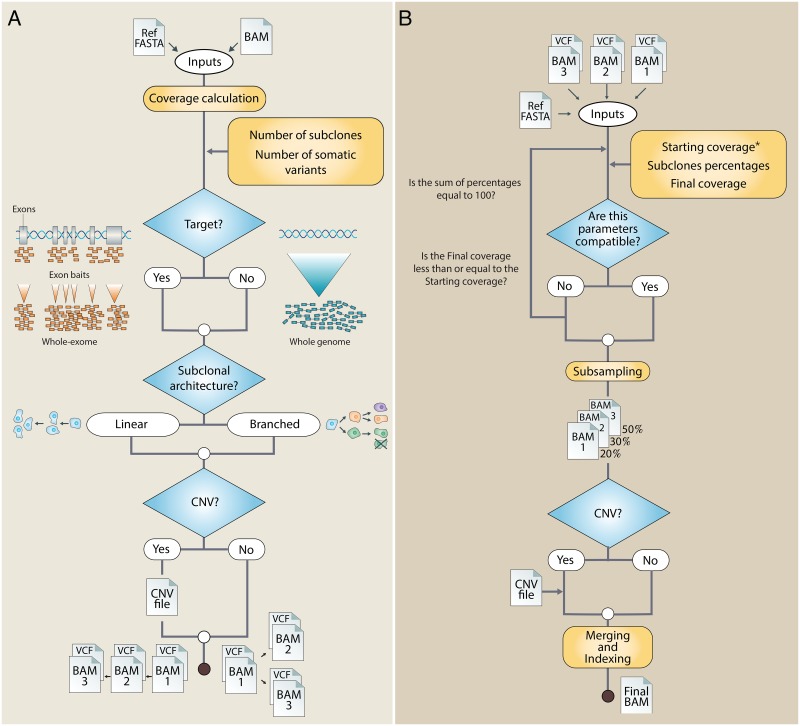
Xome-Blender work flow. Panel A shows how InXalizer works. Firstly, it calculates the input coverage, next it checks the parameters defined by the user (number of subclones, number of somatic variants and the presence or absence of target file.) and generates the subclones according to the selected subclonal architecture. Finally, if desired, it produces a CNA file. Panel B shows the Xome-Blender work flow. Firstly, it checks the parameters compatibility, next, according to the percentages defined by the user it generates subsamples of the BAM files produced by InXalizer, finally, it adds the CNA (if CNA file is provided) and merges the BAM files in the final product.

Xome-Blender is a collection of bash, R and C++ scripts based on SAMtools, Genome Analysis Tool Kit (GATK), VCFtools and Picard functions that allows to generate synthetic cancer genomes with user defined features such as the number of subclones, the number of somatic variants and the presence of CNAs, without the addition of any synthetic element. All the operations are executed by means of two bash scripts (or modules): InXalizer and Xome-Blender. The first module is devoted to the blending process initialization. It takes as input a single BAM file and basing on a set of user-defined parameters it produces a collection of alternative versions of the starting BAM that represent different tumoral clones (Fig 1).

The key idea at the base of our approach consists in starting from a normal sequenced sample (i.e. a 1000 Genome Project (1KGP) aligned experiment in BAM format) and removing known variants (SNVs and InDels) in heterozygous loci without altering the local coverage of the sequencing experiment. This is performed by editing the variants pattern of the input BAM file by using a “morphological approach” that consists in the genotype switching of a set of variants (from heterozygous to homozygous) randomly chosen among those identified by GATK in the original input genome (see Materials and methods for more details). For each selected locus, genotype switching is conducted by iteratively replacing reads that contain alternative alleles with those carrying the reference one. By using this recipe, at each step of genotype switching, the “morphological approach” moves backwards in tumor evolution process generating a clone that is the progenitor of the starting sample without adding any synthetic element. Once all clones have been generated, the Xome-Blender module subsamples them, according to the desired percentages, to generate the subclones that are, finally, merged together to produce the synthetic heterogeneous sample. Optionally, CNA events of desired size can be added. While small variants (SNVs and InDels) produce local modifications of reads, events larger than read length (≥ 100*bp*), such as copy number variants (duplications and deletions), generate an increase or decrease of coverage in duplicated or deleted regions. For this reasons, the simulations of such events need to be performed by removing or adding reads aligned to that region. Since single copy alterations arise from the deletion or duplication of a single allele, to simulate the allelic nature of CNAs the adding/removal step is performed by selecting reads with the same variants content (see Materials and methods).

### Xome-Blender evaluation

**Fig 2:**
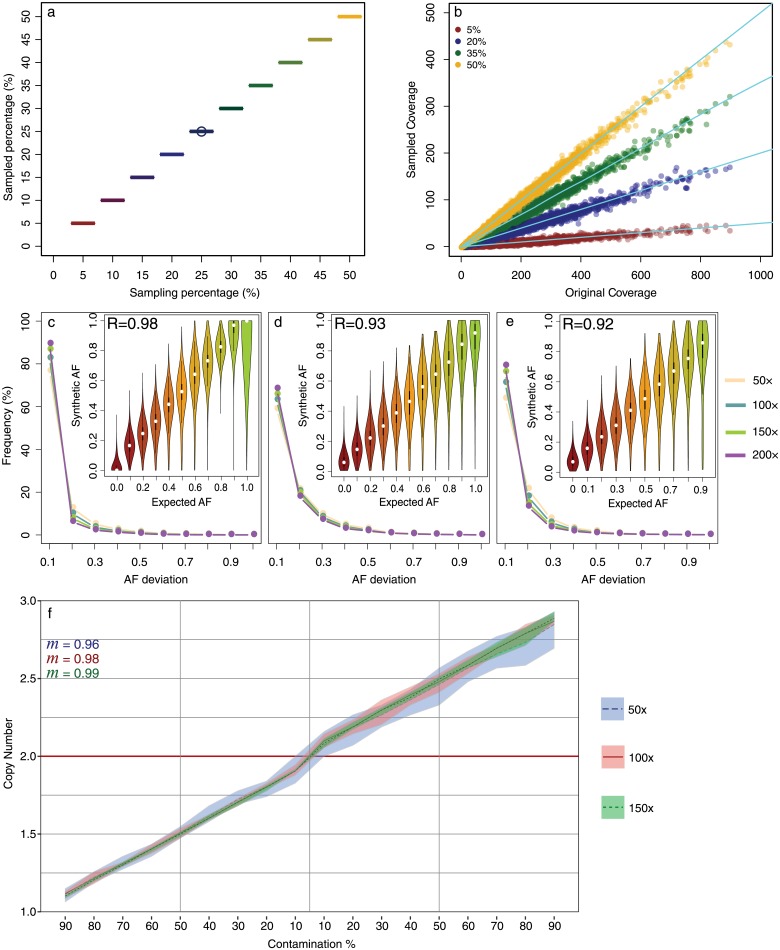
Xome-Blender evaluation. The box plot a reports the variance of the ratio between the subsample and the full-sample coverage for each different percentage. Panel B, displays the distribution of mean coverage value in the full-sample and in four different subsamples. Each data point is averaged across ten synthetic replicates. Panels C,D and E represent the Expected vs. Synthetic AF for SNVs, insertions and deletions respectively. The violin plots report the distribution of synthetic AF for bins of expected AF averaged across four coverage values (50×, 100×, 150× and 200×). The outer graphs show the distribution of the AF deviation (difference between synthetic and expected AF) averaged across the three pairs. R represent the Pearson correlation coefficient. Legend colors are referred to the average coverage of the analyzed data. Panel F represnts Expected vs. Synthtetic *log*_2_-ratio.

One of the key feature of Xome-Blender is its capability to generate tumor genomes with complex subclonal architectures by downsampling the sequencing data of normal samples with SAMtools. In order to evaluate the capability of SAMtools to correctly rescale the coverage of sequencing data, we applied the subsampling method at the base of our tool to three whole-exome seqeuncing (WES) experiments produced by the 1KGP (see Materials and methods for more details). In particular, we downsampled ten different percentages (from 5% to 50%) for each experiment and we studied coverage distributions. The results summarized in panels A-B of Fig 2 show that our downsampling strategy is capable to extract the desired percentage of reads with high accuracy without affecting the global and local coverage of the resulting sequencing data.

As a further step, to evaluate the capability of our sampling strategy to correctly preserve the AFs of small variants (SNVs and InDels), we used it to generate a set of mixed samples with different subclonal percentages and coverages. Small variants (SNVs and InDels) were selected by applying GATK HaplotypeCaller on the original WES experiments of the 1KGP and AF was estimated by means of B allele frequency (BAF), the ratio between the number of reads containing the alternative (B) allele and the total number of reads aligned to the variant position (see Materials and methods for more details).

The results reported in Fig 2C–2E and Figs A-B in S1 File show that there is high concordance between synthetic and expected AF for all the three variant classes, with SNVs that obtained the best Pearson correlation coefficient (*R* = 0.98) followed by insertions (*R* = 0.93) and deletions (*R* = 0.92). Remarkably, the absolute difference between synthetic and expected BAF is smaller 0.1 for almost 90% of SNVs and around 70% of InDels, and these results are slightly affected by sequencing coverages.

As reported in previous section, another unique feature of our tool is its capability to generate subclones with sCNAs of different size by adopting a strategy based on adding or removing reads. In order to examine the effect of the CNA-adding function, we used the third dataset and we estimated the CNA state of each sample by using the RC approach (see Materials and methods). The RC method is based on the idea that if the sequencing process is uniform (each read is sampled randomly and independently) the absolute number of DNA copies of any genomic region can be estimated by counting the number of reads that map that region.

For this reason, for each simulated alteration, we calculated the ratio between normalized RC of synthetic tumor and normal samples and we studied them as a function of sample contamination and sequencing coverage. The results reported in Fig 2F demonstrate that our CNA-adding function is capable to reproduce, with high accuracy, sCNAs at different contamination levels and the correlation with the expected copy number state increases at the increase of sequencing coverage. Remarkably, Fig 2F also reveals the different accuracy (higher variance) in the simulation of genomic regions involved in deleted or duplicated events and this can be mainly ascribed to the fact that the variance of RC data is lower for deleted states (zero or one copy) and it proportionally increases with copy number values. Taken as a whole, these results highlight how our tool is able to combine different sequencing data in any proportion, producing mixed genomes with the expected AF and CNA states, exploitable as heterogeneous samples for several kinds of applications, such as the simulation of the subclonal architecture of cancer genomes.

### Somatic callers comparison

In order to demonstrate the usefulness of Xome-Blender we used it to address the hard challenge of evaluating the performance of several state-of-the-art small variants and sCNAs calling methods in WES experiments.

The identification of somatic variants is challenging due to multiple factors. First, the sequencing coverage is non-uniform across targeted regions and between different samples [26–28], second, repetitive and paralogous sequences can originate numerous false positives and third, regions with high read depth (more than 100×) can confound variant callers and depth-based filters if not properly addressed [29]. Moreover, the genomes of primary tumors are genetically heterogeneous [30], with frequent rearrangements [31], copy number variations [32] and rare somatic mutations representing less than the 0.1% of the total variants [33–35].

#### SNV callers

**Fig 3:**
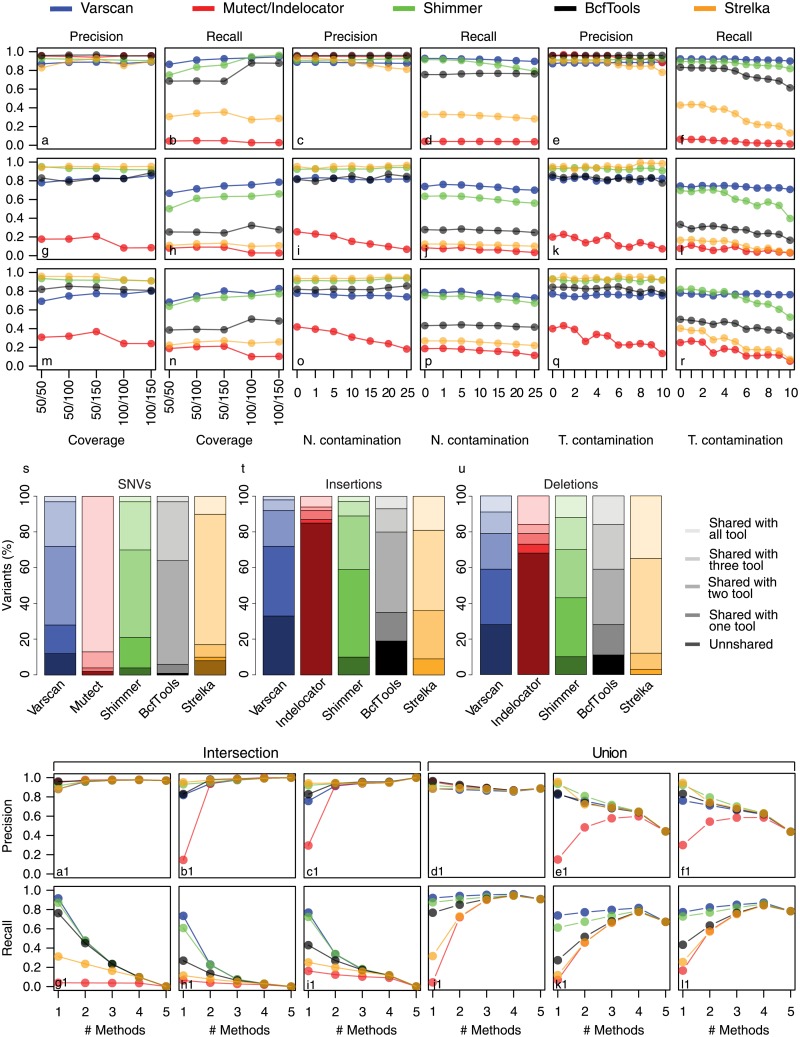
Methods performance. Panels A-R represents Precision and Recall as a function of coverages and contaminations. Panels A-F contains the SNVs data, G-L insertions data and M-R deletions data. Panels A-M, C-O and E-Q represent the precision as a function of coverages, normal contamination and tumor contamination respectively. Panels B-N, D-P and F-R represent the recall. The barplots S, T and U represent the percentage of shared or unshared variants detected by each calling method. The data are averaged across four coverage values (50×, 100×, 150× and 200×). Panels A1-L1 represent Precision and Recall for intersection and union of methods. The six boxes in the top of the Figure represent the precision. The boxes below represent the recall. Panels A1-G1/D1-J1, B1-H1/E1-K1 and C1-I1/F1-L1 contain SNVs, insertions and deletions data respectively for intersection/union.

To evaluate the performance of five widely used small variant callers (Shimmer, BcfTools, Strelka, VarScan2 and MuTect/Indelocator) with respect to different cancer sample complexity, we created a dataset of matched normal-tumor samples with different contamination levels (Fig 3). In particular, by using three 1KGP WES experiments we simulated a dataset of normal samples with ten different tumor contaminations (0%-10%) and a dataset of tumor samples contaminated with increasing level of normal tissue (0%-25%) and with 5000 somatic variants (for more details, see Materials and methods). Tumor data were simulated at sequencing coverage of 50×, 100× and 150×, while normal data at 50× and 100×. Somatic variant callers performance for SNVs, insertions and deletions were evaluated in terms of precision and recall on the following combinations of normal-tumor coverages: 50× vs 50×, 50× vs 100×, 50× vs 150×, 100× vs 100× and 100× vs 150×. Precision was calculated as the ratio between the number of correctly detected events and the total number of events detected by a tool. Recall was calculated as the ratio between the number of correctly detected events and the total number of events in the true positive set. Globally, the results summarized in panels A-R of Fig 3 show that, for both SNVs and InDels, sample contamination and sequencing coverage have a weak effect on precision (panels A-M, C-O and E-Q) and a slight influence on recall, particularly on methods that do not allow to model the sample purity, such as Shimmer, BcfTools and Strelka (panels D-P and F-R). As expected, the higher the sequencing coverage and the higher the capability of somatic caller to detect all the true positive events. In the same way, the higher the tumor contamination (the percentage of normal tissue in tumor biopsy, panels F-R) and the smaller the recall. A deeper look at these results also shows that the performance of Bayesian methods (MuTect, BcfTools and Strelka), in InDels calling, are strongly influenced by tumor contamination (panels L-R).

Surprisingly, control contamination (the percentage of tumor tissue in control sample) has weak effect on both precision and recall (panels C-O and D-P) for all the five callers, except for Indelocator that undergoes a considerable loss of precision in any simulation. Our analyses also show that VarScan2 obtains the best results in terms of precision and recall, followed by Shimmer, BcfTools, Strelka and MuTect/Indelocator. In particular, in the great majority of our analyses, Fisher’s exact test based methods (VarScan2 and Shimmer) have the best combination of precision and recall, contrary to BcfTools, Strelka and MuTect/Indelocator that show high precision at the expenses of lower recall (expecially for SNVs). These results can be partly explained by the total number of events called by each method (Table A in S1 File). Table A in S1 File and Fig 3A–3R show that the number of detected events and recall rate are highly correlated, thus demonstrating that the poor performance of MuTect and Strelka (recall) are principally due to the fact that they identified a small number of variants with respect to the true positive events generated by Xome-Blender. For these reasons we decided to study the reciprocal overlap between the calls of the five methods (Fig 3S–3U). Around 80% of SNV identified by BcfTools, Strelka and MuTect are shared by more than two callers, suggesting that these methods mainly detect “highly confident” variants (Fig 3S).

Nearly the same results are obtained for InDels detected by BcfTools and Strelka (Fig 3T and 3U). The BROAD InDel caller (Indelocator) appears poorly performant compared to its SNVs counterpart (MuTect), because the module used for InDel calling is not part of the distribution of MuTect at the time of these analyses.

The variants detected by VarScan2 are shared by two, three, four and five methods in similar proportions. This result reflects a remarkable accuracy in the detection of both hard-to- and easy-to-find variants.

As a final step, in order to understand if the combination of different tool can improve global performance, we studied the precision and recall of union or intersection of variants detected by each caller. To this end, we intersected or merged callers results in all the combination of two, three, four and five methods. A summary of these analyses is reported in Fig 3A1–3L1 and Figs C, D, E and F in S1 File. The most noticeable aspect that emerges from panels A1-L1 is the different effect produced by intersection and union.

In fact, the precision of the tools is positively influenced by intersection (Fig 3A1–3C1) contrary to the recall that undergoes a considerable reduction (Fig 3G1–3I1). On the other hand, the union of callers increase recall (Fig 3J1–3L1) and decrease precision (Fig 3D1–3F1). In particular, the effect of union and intersection is highly correlated with the performance of each method. Intersection has little effect on the precision of sensitive methods and large effect on precision of specific methods, while union has a reversed effect, increasing the recall of poorly specific callers and reducing the precision of the highly sensitive one.

A closer look at these results also shows that the best performance are obtained by combining VarScan2 and Shimmer by means of intersection (Fig C in S1 File). The other paired combinations give poor results, and this is also valid for intersection and union of three, four and five methods.

#### CNA callers

**Fig 4:**
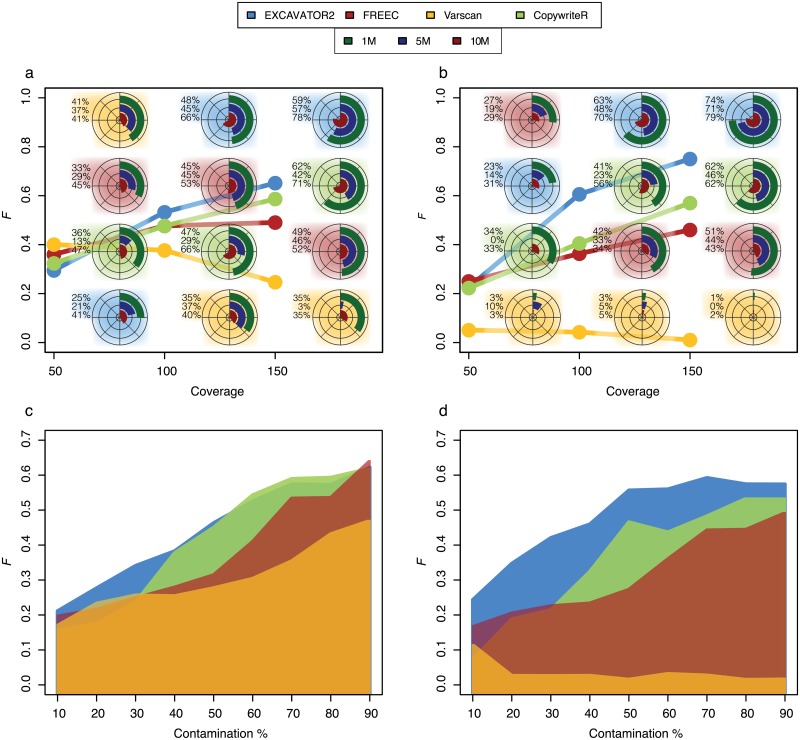
Harmonic mean of precision and recall (*F*-measure) as a function of coverage, contamination and CNA size. Panels A-C contains the insertions data and B-D deletions data. The circular barplots in panels A-B represent the *F*-score for detecting CNA of different size (1Mb, 5Mb and 10Mb) at different coverage values. The background color represent the method.

As final step, in order to test the performance of four widely used sCNAs callers (EXCAVATOR2, CopywriteR, Control-FREEC and VarScan2), we generated samples containing different spanned CNA events (1Mb, 5Mb, 10Mb). We evaluated their performance as a function of coverage (panels A-B) and contamination (panels C-D). On the whole, the results of Fig 4 show that both coverage and contamination have a considerable effect on the sensitivity of tested methods.

More precisely, the increases of these parameters lead to a gain of sensitivity, expressed as the harmonic mean of precision and recall (*F*-measure). As observed before, these methods are more sensible to deletion events (panels B-D) than duplication one (panels A-C) and the best calling performance are obtained by EXCAVATOR2 followed by CopywriteR, Control-FREEC and VarScan2.

As expected, large-sized events (10Mb) are more easily detectable than smaller one (1Mb, 5Mb) and the ability to correctly identify these events improves with increasing coverage. Taken as a whole, these results demonstrate the usefulness of Xome-Blender in testing the accuracy of each somatic caller and in comparing their performance in different and complex experimental settings.

## Discussion and conclusion

The adoption of NGS based methods in cancer research allowed for the investigation of the complex genetic structure of tumor samples, opening new doors that lead to the discovery of mutations responsible for disease onset and progression. The identification of these mutations, also called somatic variants, consists in finding variant alleles that are present in the tumor but not in the germline cells and is usually performed by using specifically tailored bioinformatics methods. To date, several computational approaches are available for this purpose, but the validation of the results produced by these methods it’s a difficult challenge due to multiple sources of error and the lack of fully characterized tumor samples. To overcome these drawbacks, different simulation approaches are used but they are often based on the addition of artificial mutations that mimic the complexity of genomic variations.

Aware of this, we developed a novel tool, called Xome-Blender, useful to generate synthetic tumor samples devoid of artificial mutations. Our pipeline takes advantage from the usage of SAMtools, GATK, Picard and VCFtools which, besides being very widespread methods, act as a collection of functions that integrate with our scripts. The simulations made with our tool showed that it is able to produce complex and heterogeneous samples preserving the intrinsic features of real sequencing data. To prove its usefulness, we generated thousands of synthetic samples collected in a dataset used to compare the performance of 5 SNV and 4 CNA widely used somatic variants calling methods. Through these analyses we observed that by using Xome-Blender data it is possible to appraise small differences between their performance and we have designated VarScan2 and EXCAVATOR2 as best tool for this kind of applications.

In conclusion, our software is a useful tool for the creation of mixed samples exploitable for the benchmarking of novel somatic calling methods and subclonal architecture reconstruction algorithms.

At present, we are working on developing and evaluating methods for subclonal architecture reconstruction starting from a dataset made of Xome-Blender samples.

## Materials and methods

### Ngs data

In this paper we used a total of four WES experiments produced by the 1KGP Consortium [36]: two samples of Caucasian (CEU) ancestry (NA12878, NA12889) and two of Yoruba (YRI) ancestry (NA18489, NA18501).

For all samples, whole-exome capture was performed by using the Agilent SureSelect All Exon V2 kit and sequencing was made by using the Illumina HiSeq2000 platform. All the reads of the four samples were aligned against the Human reference genome hg19 by means of the Burrows Wheeler aligner (BWA) [37]. The BAM file of each sample was processed, sorted and filtered (discarding MQ < 10) with SAMtools [15] and PCR duplicates were removed with Picard MarkDuplicates [38]. After duplicate removal local realignment around InDels was performed using GATK [5]. The whole-exome data were obtained for all the samples in the form of BAM alignment files from the 1KGP repository [39]. Average coverage of selected file was 150×, 193×, 71×, and 168×, for NA12878, NA12889, NA18489 and NA18501 respectively.

### Evaluation datasets

For the Xome-Blender evaluation we generated three different datasets. The first one was useful to study the subsampling method and it’s effect on samples coverage. To this end, we subsampled three 1KGP WES experiments (NA12878, NA18489, NA18501) at ten different percentages: 5%, 10%, 15%, 20%, 25%, 30%, 35%, 40%, 45% and 50%. Each percentage was replicated ten times. Subsampling was performed by using samtools view -s command of SAMtools v1.2, downloadable at http://www.htslib.org.

The second dataset was used to evaluate the effect of subsampling on AF. In particular, we simulated synthetic matched normal-tumor samples with 10-90%, 20-80%, 30-70%, 40-60% and 50-50% proportions, at four different coverages (50×, 100×, 150× and 200×). To calculate the synthetic BAF we first used SAMtools [15] to generate the mpileups of each BAM file and we then applied pileup2base [40] to manipulate the information contained in the mpileup files. On the other hand, expected BAF was calculated by using reference and alternate allele estimated by GATK HaplotypeCaller v3.3. Finally, in order to examine the effect of the CNA-adding function, we produced a third dataset of matched normal-tumor samples, containing sCNAs events of variable size (1Mb, 5Mb and 10Mb) and at increasing contamination level (from 10% to 90% of tumor contamination). For each synthetic dataset we generated replicates at 50×, 100× and 150× of sequencing coverage and we then estimated the CNA state by using the RC approach.

### Variant calling benchmark dataset

To test the performance of the five small somatic variants calling methods, we created a dataset of matched normal-tumor samples reflecting different contamination levels. In particular, by using the three higher coverage WES experiments (NA12878, NA12889, NA18501), we simulated a dataset of normal samples with ten different tumor contaminations (0%, 1%, 2%, 3%, 4%, 5%, 6%, 7%, 8%, 9% and 10%) and a dataset of tumor samples contaminated with increasing level of normal tissue (0%, 1%, 5%, 10%, 15%, 20% and 25%) and with 5000 somatic variants, including 4778 SNVs and 222 InDels (97 Insertions and 125 Deletions). Tumor data were simulated at sequencing coverage of 50×, 100× and 150×, while normal data at 50× and 100×. Somatic variant callers performance for SNVs, insertions and deletions were evaluated in terms of precision and recall on the following combinations of normal-tumor coverages: 50× vs 50×, 50× vs 100×, 50× vs 150×, 100× vs 100× and 100× vs 150×. Precision was calculated as the ratio between the number of correctly detected events and the total number of events detected by a tool. Recall was calculated as the ratio between the number of correctly detected events and the total number of events in the true positive set. To test the CNA callers we used the same dataset used to make some tests on the CNA adding function (see Evaluation datasets).

### Calling methods

The synthetic datasets described in previous section (Variant calling benchmark dataset) were used to compare sensitivity and specificity of several state-of-the-art small variants and sCNAs calling methods in WES experiments. To compare results of different callers the vcf files produced by each method were processed with home-made scripts.

#### Small somatic variants callers

The compared methods for small somatic variants are the following (for more details on parameters configurations see Supplemental Materials):

**VarScan2** takes as input the pileup files from the tumor and normal samples and analyzes them independently and calls a genotype for each position that reaches predefined thresholds of coverage and quality. Each position of the genome is classified into somatic, germline, or ambiguous by applying the Fisher’s exact test on the number of reads containing variants in tumor and normal samples [13].
**MuTect** takes as input the normal and the tumor BAM files and detects only somatic point mutations using a Bayesian classifier approach. The method first analyzes separately the aligned reads in tumor and normal samples and than post-process the resulting variants by applying an additional set of filters [12].
  To call insertions and deletions we used Indelocator that isn’t part of the current MuTect distribution. In its default mode, Indelocator uses the same inputs of MuTect. Without performing realignment or split-read alignment, the tool looks for events present in tumor sample above a specified thresholds and having sufficient coverage in normal sample. Recorded mutations are also annotated as (putatively) germline or somatic depending on the presence of the alternative allele in normal sample.
**Shimmer** takes as input aligned sequence reads from a tumor and its matched normal tissue in BAM format. By examining the base counts for each possible allele at every covered genomic position in both samples, Shimmer selects sites displaying a non-reference allele over a minimum threshold. Subsequently, a Fisher’s exact test is performed to test the null hypothesis that variant alleles are distributed randomly between the two samples. By performing a multiple testing correction [41, 42] on the Fisher exact test P-values, only a set of results is reported. In particular, those with false discovery rate (FDR) below a desired maximum threshold and predicted as homozygous reference in the normal sample [28].
**BcfTools** is a set of utilities that manipulate variant calls in the VCF and its binary counterpart BCF. SAMtools/BcfTools is probably the most commonly used algorithm for calling SNV, it calls SNV genotypes independently and its likelihood function assumes no Allelic specific expression (ASE). When read counts are very low, SAMtools/BcfTools may not call SNV genotypes [15].
**Strelka** takes as input the sequencing data files of tumor and normal samples in BAM format and by applying a Bayesian probability model define the most likely genotype. Variant detection is also based on a set of built-in filters based on factors such as read depth, mismatches, and overlap with InDels.
  We skipped depth filtration for exome sequencing data as recommended by the Strelka authors [16].

#### sCNA callers

The compared sCNA calling methods are the following (for more details on parameters configurations see Supplemental Materials):

**VarScan2** identifies SCNAs by means of a three-step process, first compares BQ 20 read depths between tumor and normal samples for contiguous regions of coverage. After normalizing for the amount of input data (unique bases mapped), the relative copy number change is inferred as the log2 of the ratio of tumor depth to normal depth for each contiguous region. Then, it applies a circular binary segmentation (CBS) algorithm (Seshan and Olshen 2010) to delineate segments by copy number and identify significant change-points and finally, merges adjacent segments of similar copy number and classify them as either large-scale (>25% of chromosome arm) or focal events (<25%) [13].
**EXCAVATOR2** is based on the RC approach, unlike its previous version (EXCAVATOR), it enhances the identification of genomic CNVs (overlapping or non-overlapping exons) from WES data by integrating the analysis of In-targets and Off-targets reads. It’s workflow starts with the RC calculation followed by the correction of the data for GC-content, mappability and exon size. After normalization, normalized read count (NRC) for each sample are organized according to the analysis mode (pooling or somatic) selected by the user: pooling mode to compare one sample to a pool of normal controls, somatic mode to compare one sample to its corresponding normal control. Finally, heterogeneous shifting level model (HSLM) is applied to segment the two combined profiles and FastCall algorithm classifies each segmented region into five possible states (two-copy deletion, one-copy deletion, normal, one-copy duplication and multiple-copy amplification). The results are provided as tab-delimited text files and as Figures for raw and normalized data, plots of segmentation and calling results [8, 43].
**CopywriteR** allows for extracting uniformly distributed copy number information analysing In and Off-targets reads. It can be used without reference, and can be applied to sequencing data obtained from various techniques including chromatin immunoprecipitation and target enrichment on small gene panels [44].
  The sequencing data are analysed by means of three functions. The first function generates mappability and GC-content files for the provided bin size. The second function calculates compensated RCs, performs the mappability and GC-content-based normalization steps and applies a filter for regions of germline copy number variation. The results are provided in tab-separated format. The third function is optional and allows segmentation using CBS [45] as implemented in the R-package CGHcall 2.22.0 [46], and plotting of the results.
**Control-FREEC** takes as an input aligned reads, then constructs and normalizes the copy number profile, constructs the BAF profile, segments both profiles, ascribes the genotype status to each segment using both copy number and allelic frequency information, then annotates genomic alterations. If a control (matched normal) sample is available, Control-FREEC discerns somatic variants from germline ones [47].

### Xome-Blender

The first module of Xome-Blender takes as input a single BAM file and basing on a set of user-defined parameters it produces a collection of alternative versions of the starting BAM, representing clones, and their respective VCF file.

Moreover, InXalizer calculates the coverage of the input file by using the samtools depth function and optionally creates a file containing the coordinates to insert CNA in the final product. The initialization can be tuned by means of four parameters:

- Subclone number = the number of subclones that will compose the final product.
- Variants number = the number of somatic variants that will appear in the final product.
- Subclonal architecture = the evolution model for the sample synthesis, it can be linear or branched.
- CNV = it’s an option that allows for the generation of a CNA file, defining their number and length.

In order to generate the different clones, a label and a fasta reference sequence are required. Optionally, it is possible to use a target file, in bed format, to edit only defined portions of the BAM file (whole-exome or target sequencing experiments). The InXalizer’s outputs are then used as inputs for the second module, Xome-Blender. The first step of its workflow consists in parameter checking. In fact, as for InXalizer, it is possible to drive the blending process by setting two variables:

- Percentages = the desired percentage of each subclone (including the control).
- Final coverage = the average coverage of the final product.

If the values of these parameters are incompatible with the input data features (for example the “starting coverage” stored in the *.cov file), the process stops. Alternatively, it goes to the next step and it starts to subsample the clone BAM files (generated by InXalizer) to get the desired percentage of each of these and next it adds the CNA events, if a CNA file is provided.

The CNA adding function operates on each clone allowing to generate clone-specific events. In order to make a CNA, firstly, a BAM file of the interested region is generated. Next, all its variants are identified and the alternative reads aligned on these positions are stored in a new BAM file. Contemporary, a file containing the reference reads is generated and randomly subsampled at 50%. Both BAM are then sorted.

In case of deletion, it is necessary to proceed with a further step which involves the storing of all reads-in-region in a file common for all deletion events. At the end of the process all these reads are removed from the final product, producing deletions.

The resulting BAM files are then merged and indexed by means of the samtools merge and index functions.

## Supporting information

S1 File Supplemental PDF file containing parameters configurations of calling methods, supplemental figures and supplemental tables. (PDF)
