# Supplementary material for: Xome-Blender: A novel cancer genome simulator
Source: PLoS One. 2018 Apr 5;13(4):e0194472. doi: 10.1371/journal.pone.0194472 (PMC5886411; doi:10.1371/journal.pone.0194472)
Supplement: S1 File — (PDF) [file pone.0194472.s001.pdf]

Supplemental Materials to "Xome-Blender: a novel cancer genome simulator."

Roberto Semeraro, Valerio Orlandini, Alberto Magi

1 SUPPLEMENTAL METHODS

2 Parameters configuration

2.1 SNV callers

VarScan2 We first generated the mpilup file:

```
samtools mpileup -B -q 1 -f ref.fa tumor.bam normal.bam >
tumor-normal.mpileup
```

Then, in order to increase the detection sensitivity we did run VarScan2 on each sample calculating `normal-purity` and `tumor-purity` on the base of the sample composition (proportions):

```
VarScan somatic tumor-normal.mpileup file.out
--mpileup 1 \
--normal-purity \
--tumor-purity \
--strand-filter 1
```

Finally, we filtered the output file with processSomatic:

```
VarScan processSomatic file.out.SNV
VarScan processSomatic file.out.indel
```

MuTect In order to increase the detection sensitivity, for each sample we calculated `minimum normal allele fraction` and `minimum mutation cell fraction` on the base of the sample composition (proportions).

```
muTect --analysis_type MuTect --reference_sequence ref.fa \
--input_file:normal norm.bam \
--input_file:tumor tum.bam \
--dbSNV dbSNV.vcf \
--minimum_mutation_cell_fraction \
--minimum_normal_allele_fraction \
--only_passing_calls \
--out call_stats.out \
--coverage_file coverage.wig.txt \
--vcf MutectOut.vcf
```

Indelocator

```
Indelocator --analysis_type IndelGenotyperV2 \
--somatic \
--reference_sequence ref.fa \
--input_file:normal norm.bam \
--input_file:tumor tum.bam \
--out somatic.indels
```

Shimmer

```
Shimmer norm.bam tum.bam --ref ref.fa
```

BcfTools For BcfTools we used samtools-0.1.19

```
samtools mpileup -DSuf ref.fa norm.bam tum.bam | bcftools
view -bvcgT pair - | bcftools view - > output.vcf
```

Strelka

```
configureStrelkaWorkflow.pl \
--normal=norm.bam \
--tumor=tum.bam \
--ref=ref.fa \
--config=config.ini

make -j 1
```

2.2 CNV callers

VarScan2

```
samtools mpileup -B -q 1 -f ref.fa -l region.bed tum.bam \
norm.bam | awk -F ’“‘t’’‘$4 > 0 && $7 > 0’| VarScan \
copynumber - OutputName --mpileup 1
VarScan copyCaller OutputName.copynumber --output-file
```

EXCAVATOR2

```
perl EXCAVATORDataPrepare.pl ExpFilePrepare.w20000.txt \
--target MyTarget.20000 \
--assembly hg19
perl EXCAVATORDataAnalysis.pl ExpFileAnalysis.w20000.txt \
--target MyTarget.20000 \
--assembly hg19 \
--mode paired
```

CopywriteR We generated the R script as suggested by authors.

```
> library(CopywriteR)
> preCopywriteR(output.folder = file.path(’./path/out/’),
bin.size = 20000,
```

```
ref.genome = ''hg19'',
prefix ='''')
> bp.param <- SnowParam(workers = 1,
type = ''SOCK'')
> sample.control <- data.frame(samples, controls)
> CopywriteR(sample.control = sample.control,
destination.folder = file.path(''/path/to/''),
reference.folder = file.path(''./path/to/'', ''hg19_20kb''),
bp.param = bp.param)
> plotCNA(destination.folder = file.path(data.folder))
```

Control-FREEC We created a config file for each experiment, using a calling window of 20kb.

```
./freec -conf config.file
```

3 Supplemental Figures

Here we proof the correlation between synthetic and expected AF and the performance obtained by intersection or merging of callers results in all the combination of two, three, four and five methods.

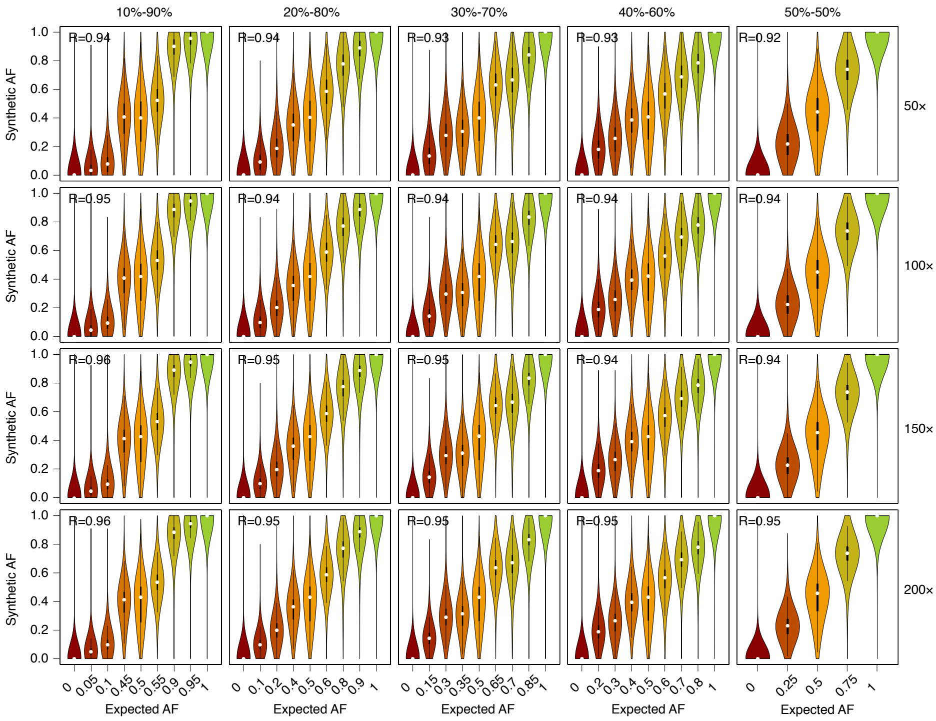

Figure A: Expected vs. Synthetic AF for SNVs. Each violin report the distribution of synthetic AF for bins of expected AF. For each panel the values on the top and on the right represent the normal-tumor proportion of the sample and its coverage respectively. R represent the Pearson correlation coefficient.

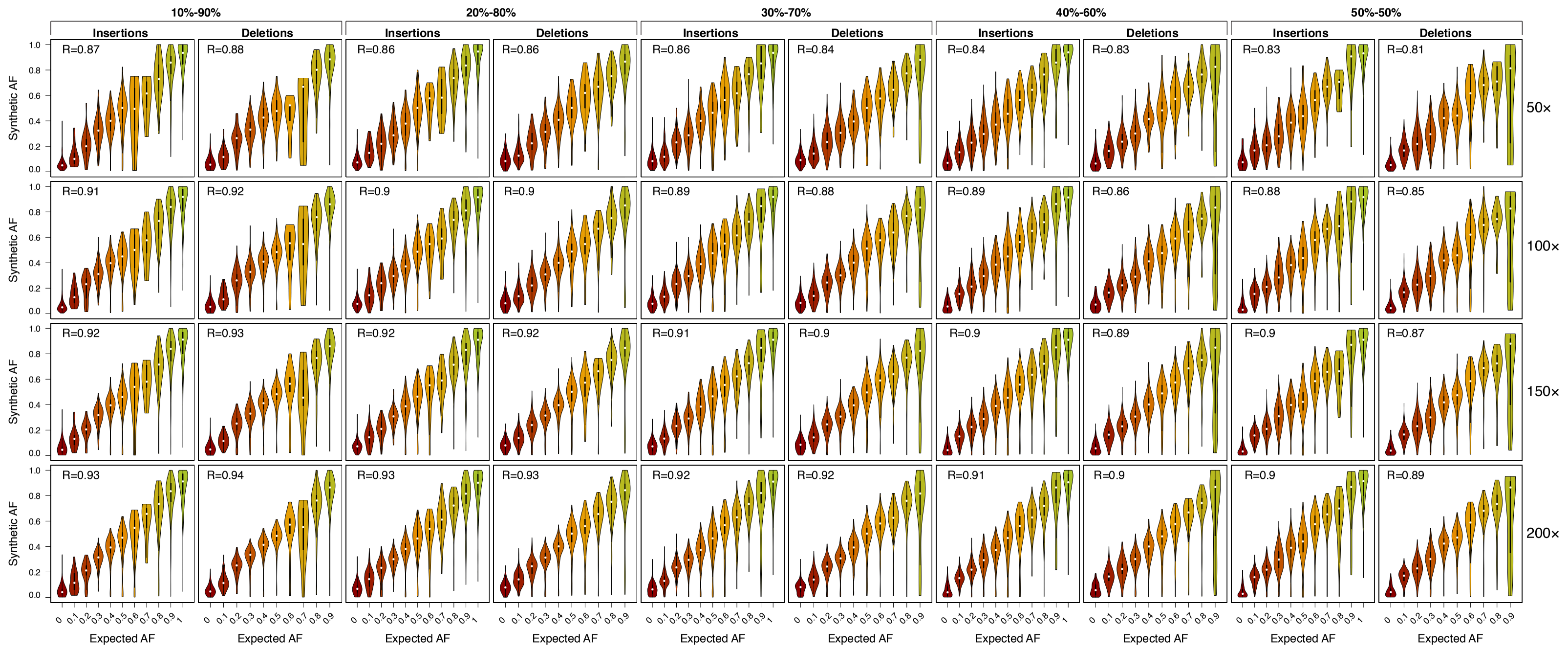

Figure B: Expected vs. Synthetic AF for insertions and deletions. Each violin report the distribution of synthetic AF for bins of expected AF. For each panel the values on the top and on the right represent the normal-tumor proportion of the sample and its coverage respectively. R represent the Pearson correlation coefficient.

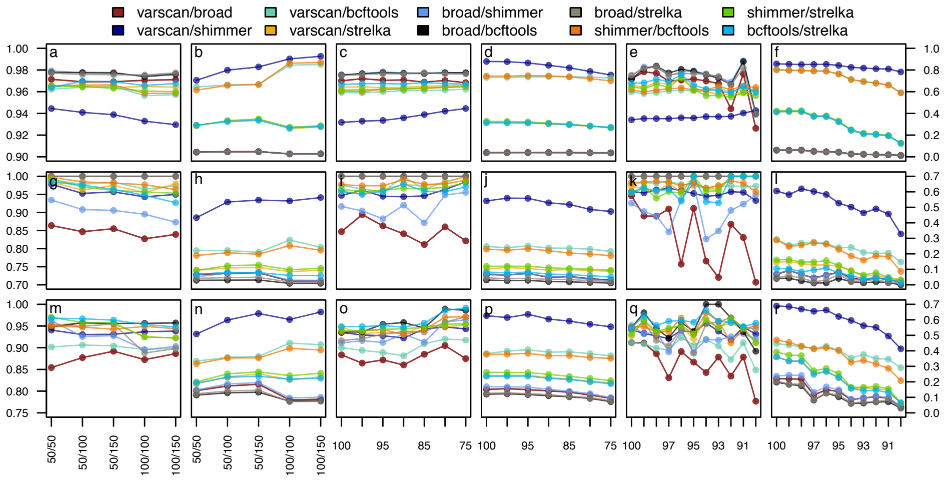

Figure C: Precision and Recall of intersections of pairs of methods as a function of coverages and contaminations. Panels a-f, g-l and m-r contain SNVs, insertions and deletions data respectively. Panel a-m, c-o and e-q represent the precision as a function of coverages, normal contamination and tumor contamination respectively. Panel b-n, d-p and f-r represent the recall. Labels on the left and right represent precision and recall values respectively.

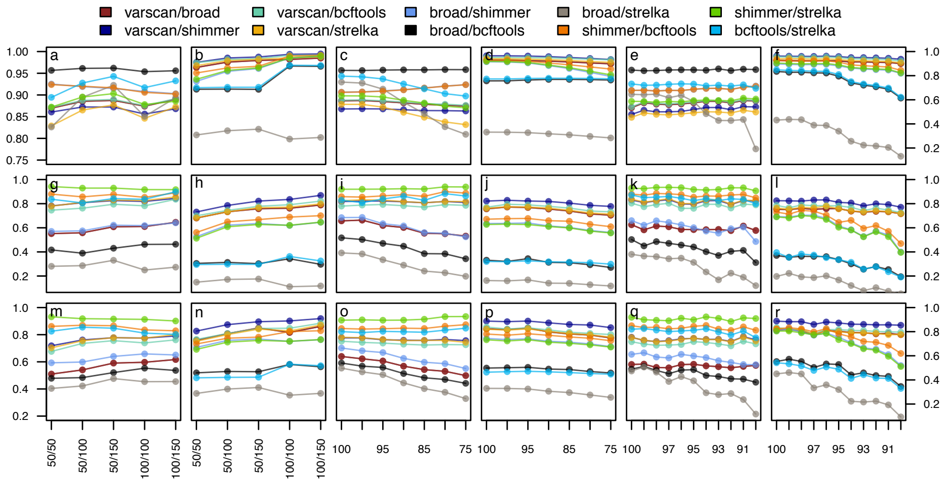

Figure D: Precision and Recall of unions of pairs of methods as a function of coverages and contaminations. Panel description is the same of figure C.

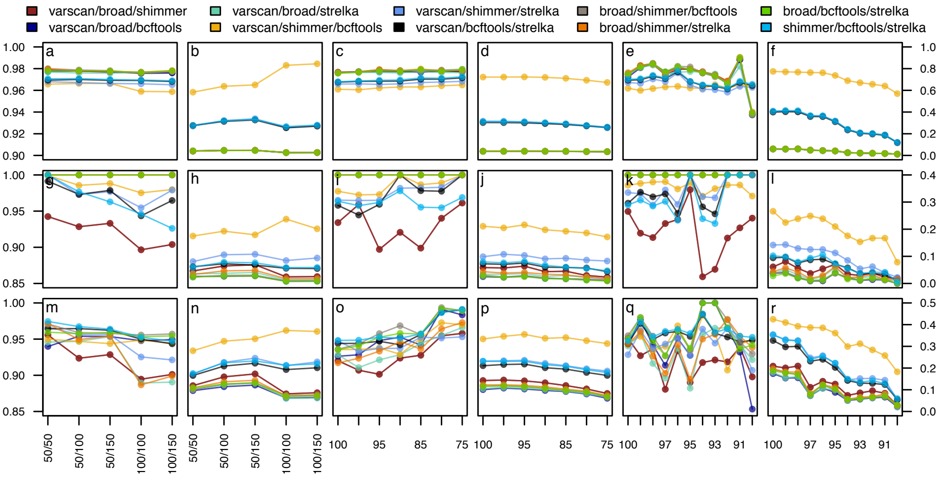

Figure E: Precision and Recall of intersections of three methods as a function of coverages and contaminations. Panel description is the same of figure C.

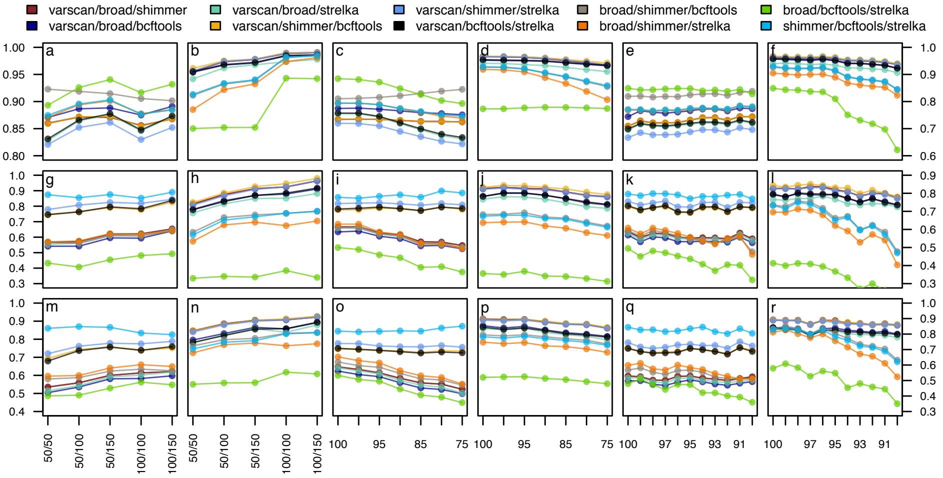

Figure F: Precision and Recall of unions of three methods as a function of coverages and contaminations. Panel description is the same of figure C.

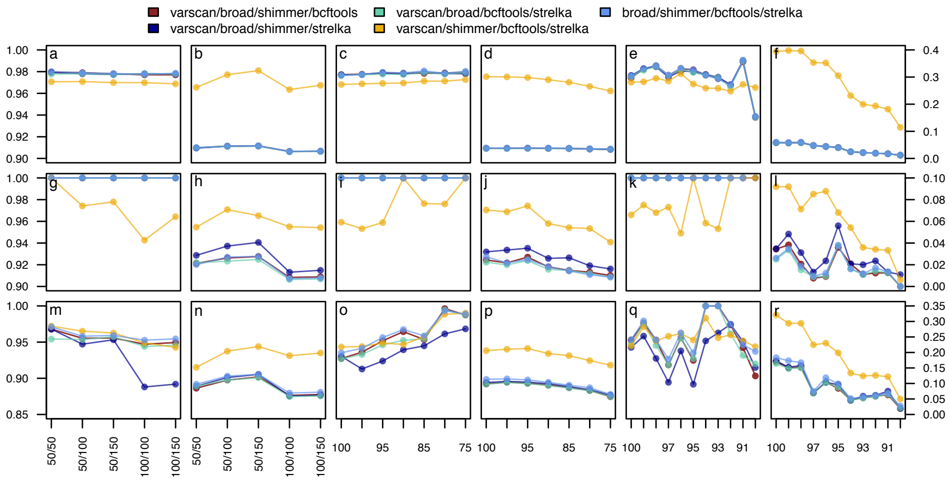

Figure G: Precision and Recall of intersections of four methods as a function of coverages and contaminations. Panel description is the same of figure C.

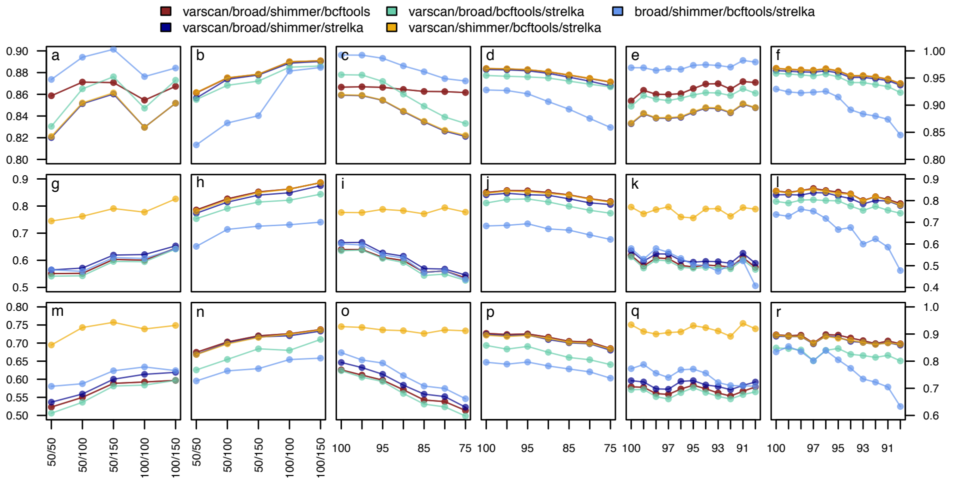

Figure H: Precision and Recall of unions of four methods as a function of coverages and contaminations. Panel description is the same of figure C.

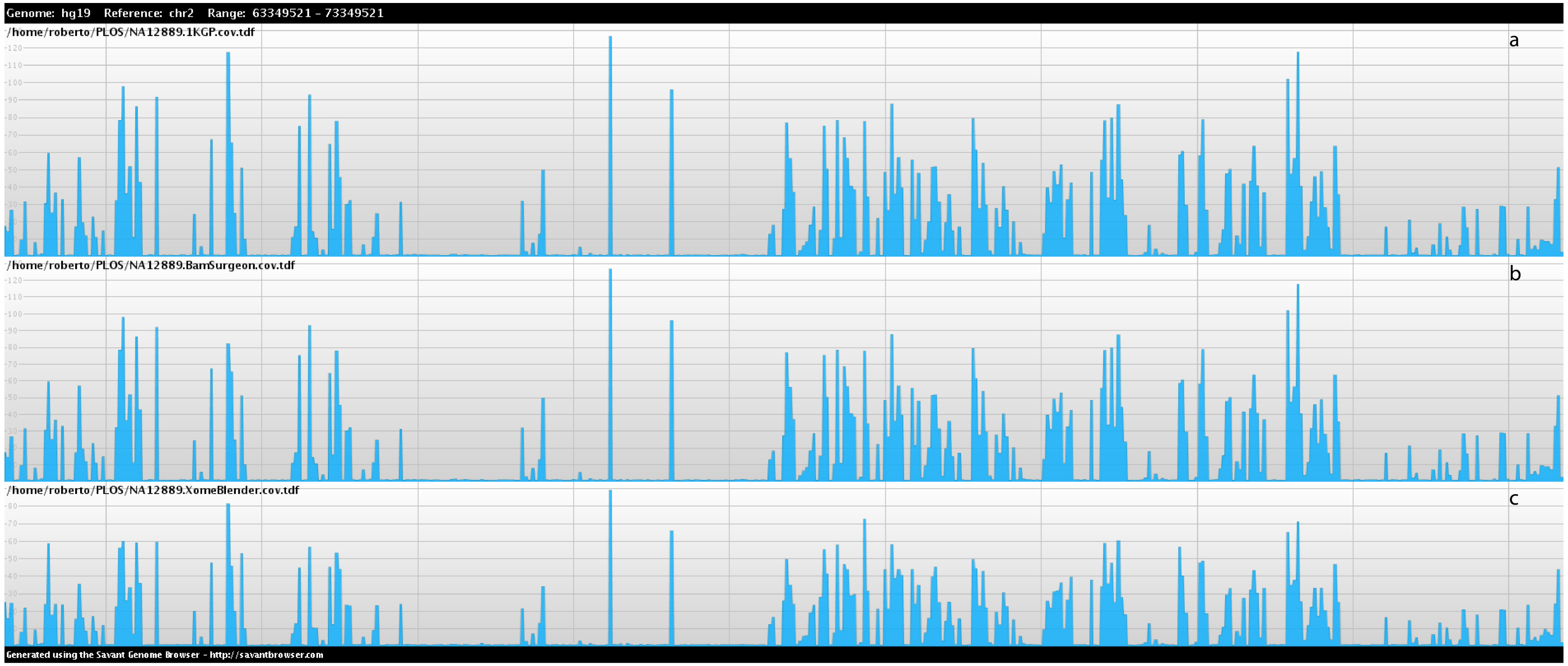

Figure I: Coverage comparison of deletions generated by BamSurgeon and Xome-Blender. Panels b and c report the sequencing coverage generated by BAMSurgeon and Xome-Blender to simulate a 10 Mb deletion. Panel a reports the sequencing coverage of the same 10 Mb region for the normal sample. The three panels clearly demonstrate that while our approach is capable to reduce coverage, generating genuine deletions, BamSurgeon do not affect the sequencing coverage of the region.

4 Supplementale Tables

Table A: Number of SNVs, Insertions and Deletions detected by each method at different normal-tumor coverage combinations.

	**Coverage**														
	**50/50**			**50/100**			**50/150**			**100/100**			**100/150**		
VarScan2	4751	83	123	4908	86	125	4972	87	129	5100	89	126	5070	89	129
Shimmer	3870	51	85	4334	64	98	4476	66	100	4977	67	102	5085	70	106
BcfTools	3421	30	59	3408	31	58	3393	29	58	4403	38	77	4380	30	75
Strelka	1766	11	29	1819	13	34	1829	14	35	1534	10	33	1530	11	36
MuTect/Indellocator	227	44	76	246	50	81	249	43	72	137	35	54	141	33	54
